# The link between neutrophils, NETs, and NLRP3 inflammasomes: The dual effect of CD177 and its therapeutic potential in acute respiratory distress syndrome/acute lung injury

**DOI:** 10.17305/bb.2023.10101

**Published:** 2024-08-01

**Authors:** Jingying Li, Zhansheng Fang, Shumin Xu, Haiwei Rao, Junzhe Liu, Kunjian Lei, Lufei Yang, Chong Wang, Zhenguo Zeng

**Affiliations:** 1 Department of Critical Care Medicine, The 2nd Affiliated Hospital, Jiangxi Medical College, Nanchang University, Jiangxi, China; 2 Department of Neurosurgery, The 2nd Affiliated Hospital, Jiangxi Medical College, Nanchang University, Jiangxi, China; 3 JXHC Key Laboratory of Neurological Medicine, Jiangxi, China; 4 Department of Operating Room, The 2nd Affiliated Hospital, Jiangxi Medical College, Nanchang University, Jiangxi, China; 5 Department of Critical Care Medicine, The 1st Affiliated Hospital, Jiangxi Medical College, Nanchang University, Jiangxi, China

**Keywords:** Acute respiratory distress syndrome (ARDS), CD177, NLRP3, neutrophil extracellular traps (NETs), reactive oxygen species (ROS).

## Abstract

Neutrophils are important inflammatory effector cells that protect against foreign invasion but also cause self-harm. Numerous neutrophils infiltrate the lungs in acute respiratory distress syndrome/acute lung injury (ARDS/ALI) patients. However, the exact impact of neutrophil infiltration on ARDS’s onset and progression remains unclear. To investigate this, we analyzed two ARDS-related datasets from the Gene Expression Omnibus public database and discovered an association between CD177, a neutrophil-specific surface protein, and ARDS progression. We used quantitative flow cytometry to assess CD177+ neutrophils in the peripheral blood of clinical ARDS patients vs healthy controls, finding a significant increase in CD177+ neutrophils percentage among total neutrophils in ARDS patients. This finding was further confirmed in ALI mouse models. Subsequent animal experiments showed that anti-CD177 effectively reduces pulmonary edema, neutrophil infiltration, and inflammatory cytokine release, along with a decrease in reactive oxygen species (ROS) and myeloperoxidase (MPO) levels. We also established an in vitro co-culture system to mimic neutrophil and lung epithelial cell interactions. In the anti-CD177 group, we observed decreased expression of NLRP3, caspase 1, peptidyl arginine deiminase (PAD4), MPO, and ROS, along with a reduction in certain inflammatory cytokines. These results indicate a crucial role for the CD177 gene in ARDS’s development and progression. Inhibiting CD177 may help mitigate excessive activation of NLRP3 inflammasomes, ROS, and neutrophil extracellular traps (NETs), thus alleviating ARDS.

## Introduction

Acute respiratory distress syndrome (ARDS) is a severe lung tissue injury caused by various factors, leading to diffuse alveolar damage and the infiltration of diverse inflammatory cells. This results in impaired alveolar gas exchange, restricting oxygen entry into the blood circulation and causing severe hypoxia [1, 2]. The inflammatory pathogenesis theory is widely accepted in the academic community [3]. External stimuli mediate the infiltration of inflammatory cells into lung tissue, and their indiscriminate attack on pulmonary blood vessels and alveolar tissues is the primary cause of respiratory distress in ARDS patients [4, 5]. Regulating the infiltration of inflammatory cells, prompted by external factors through gene regulation, can significantly reduce pulmonary edema, limit immune cell infiltration, enhance the effectiveness of drugs and supportive therapies for ARDS treatment, and significantly improve the prognosis of ARDS patients [6]. Consequently, our study aims to modulate the infiltration of various inflammatory cells in lung tissue, alter the expression of characteristic molecules affecting cell signal transduction on their surfaces, reduce the infiltration of inflammatory cells, mitigate the uncontrolled inflammatory response, alleviate overall damage to ARDS-affected lung tissue, and effectively enhance the prognosis of ARDS patients [7, 8].

Previous studies have established extensive neutrophil infiltration in the lung tissues of ARDS patients [9, 10]. By integrating data from multiple public databases on inflammatory patients, we identified CD177 as a molecule differentially expressed in ARDS patients, closely associated with their poor prognosis. In line with prior studies [11–13], we found that modulating CD177 expression levels on the neutrophil surfaces significantly impacts their migration, reactive oxygen species (ROS) release, and neutrophil extracellular trap (NET) formation. Our results also suggest that CD177 regulates the activation of the NLRP3 inflammasome. This discovery highlights the dual role and mechanism of CD177 in ARDS, which contrasts with its previously reported inflammatory protective effect [11]. CD177 appears to have a positive feedback effect in the development and progression of ARDS.

## Materials and methods

### Data acquisition and exclusion criteria

Microarray datasets were procured from the Gene Expression Omnibus (GEO; http://www.ncbi.nlm.nih.gov/geo) database and screened based on the following criteria: 1) inclusion of neutrophil samples from clinical ARDS patients and matched healthy volunteers, excluding samples from healthy volunteers treated with PI3K inhibitors; 2) complete clinical characteristics for ARDS patients; and 3) a minimum of 20 patients and healthy controls in the study sample. Based on these criteria, two datasets—GSE76293 and GSE172114—were selected for further analysis. Specifically, the GSE76293 dataset comprised neutrophil samples from 12 clinical ARDS patients and 12 healthy volunteers, while the GSE172114 dataset included 22 healthy controls and 47 clinical ARDS patients. Differentially expressed gene (DEG) filtering was performed on both datasets to identify common DEGs, and WGCNA analysis was separately conducted on the GSE76293 dataset.

### Target genes associated with ARDS were screened based on bioinformatics analysis

Utilizing the R package “limma,” DEGs between neutrophil samples from ARDS patients and matched healthy volunteers were identified, applying the criteria of *P* < 0.01 and |log_2_(fold change)|>1 in the GSE76293 and GSE172114 cohorts. In the GSE76293 cohort, 189 downregulated and 209 upregulated genes were identified as DEGs. Gene ontology (GO) and Kyoto Encyclopedia of Genes and Genomes (KEGG) analyses were performed using the R package “clusterProfiler.” A Venn diagram identified 39 overlapping DEGs, and five genes (*CD177*, *TDRD9*, *GPR84*, *CYP1B1*, and *MCEMP1*) showing greater variability in both cohorts were selected. WGCNA analysis, performed by using the “WGCNA” R package, identified modules with the most clinically relevant features, and their biological functions were further explored through GO and KEGG analysis. For immune cell profiling, the CIBERSORTx website (https://cibersortx.stanford.edu/) was used. This tool calculates gene expression profiles and estimates the abundance of member cell types in a mixed cell population. It was applied to assess the fraction of 22 types of immune cells among the GSE76293 and GSE172114 samples.

### Clinical ARDS patients and blood samples

We enrolled all ARDS patients admitted to the Department of Critical Care Medicine at the Second Affiliated Hospital of Nanchang University (Nanchang, China) from June 2020 to March 2023. The inclusion criteria followed the Berlin standard for ARDS patients [14], such as intrapulmonary and extrapulmonary bacterial infections as the cause, age between 18 and 60 years, previous good health without chronic diseases or immunodeficiency, and adoption of invasive mechanical ventilation with PaO_2_/FiO_2_ ≤200 mmHg. We collected EDTA-anticoagulated blood samples (20 mL) from confirmed ARDS patients (ARDS, *N* ═ 6) and healthy controls (HC, *N* ═ 6).

### Lipopolysaccharide (LPS)-induced lung injury model

The animal subjects for this study were 8-week-old C57BL/6J mice, weighing between 20 and 30 g, obtained from GemPharmatech Co., Ltd., Nanchang, China. The mice were randomly divided into two sequences, each sequence consisting of four groups with either six or four mice per group. Throughout the experiment, the mice received daily intratracheal injections of LPS (*Escherichia coli O55:B5*, Solarbio, Beijing, China) or saline (used as a negative control). Simultaneously, intraperitoneal injections of 200 µg of CD177 monoclonal antibody (Santa Cruz, sc-374291) or IgG antibody (serving as a negative control) were administered. The LPS concentration was 2 mg/mL, with a dosage of 10-mg/kg body weight. After isoflurane anesthesia, we instilled LPS or saline into the mice’s lungs via a catheter. Following the injection, the mice were allowed to recover and returned to their cages with unrestricted access to food and water. For the first sequence of mice, euthanasia was performed 48-h post-administration, and lung tissue samples were collected under anesthesia for subsequent histological and biochemical analyses. In the case of the second sequence of mice, we administered the treatment continuously for a week and recorded their survival status and clinical manifestations. We conducted three clinical assessments daily for all mice, scoring them based on the following criteria [15]: 0 ═ normal response to stimuli; 1 ═ ruffled fur, sluggish response; 2 ═ response only to repeated stimuli; 3 ═ no response or circling; and 4 ═ death. If a mouse exhibited extreme lethargy or neurological abnormalities (score ═ 3), it was deemed moribund and humanely euthanized immediately. We performed lavage of the lungs by intubating the trachea and instilling 2 mL of sterile saline to collect bronchoalveolar lavage fluid (BALF). After the centrifugation of BALF samples, we stored the supernatants at –80 ^∘^C for subsequent cytokine measurements using enzyme-linked immunosorbent assay (ELISA) kits (Enzyme-linked Biotechnology, China).

### Processing of peripheral blood samples

Peripheral blood was collected from both humans and mice using blood collection vessels. Initially, the peripheral blood underwent moderate lysis with red blood cell lysate, and non-lysed cells were immediately collected through centrifugation. Subsequently, the human neutrophil enrichment kit (EasySep™ Human Neutrophil Enrichment Kit, Stem cell) was employed for isolation and purification.

### Flow cytometric analysis

Peripheral mononuclear cells from patients or mice, obtained through differential centrifugation, were incubated with CD66b^+^ or Ly-6G^+^ fluorescent antibodies at 4 ^∘^C for 3 h. Neutrophils positive for fluorescent antibodies were sorted using a flow cytometer (BD FACSAria, USA) and collected in a complete culture medium. Next, we labeled the neutrophils again with CD177^+^ antibodies and performed flow cytometry analysis using the CytoFLEX B3-R3-V2 flow cytometer (Beckman Coulter, USA) to assess the quantity and phenotype of the neutrophils. Data processing was performed using Flowjo_V10 software (FLOWJO, LLC; Ashland, USA). We gated human neutrophils based on CD66B+ expression and CD177^+^ neutrophils based on CD66b^+^CD177^+^ double positivity. We gated mouse neutrophils based on CD66b^+^Ly6G^+^ double positivity and further analyzed them (Figure S3). All antibodies used for flow cytometry experiment were directly labeled (IgG-FITC [BD, USA], IgG-PE [BD, USA], CD66b^+^-FITC [BD, USA], CD177^+^-PE [BD, USA], and Ly-6G^+^-FITC [BD, USA]).

### Histological examination and immunohistochemistry

Upon collection, mouse tissues were immediately fixed with 4% paraformaldehyde, followed by dehydration with alcohol, routine paraffin embedding, and made into 2-3 mm sections. These sections were then stained with hematoxylin and eosin for histopathological evaluation. For immunohistochemistry, the slides were dewaxed and rehydrated at room temperature. Antigen retrieval was carried out using citrate buffer (10 mM, pH 6.0) and the endogenous enzyme activity was quenched with 3% hydrogen peroxide. Following blocking with goat serum, the slides were incubated overnight in a humidified chamber at 4 ^∘^C with a mouse anti-human CD177 antibody (1:200; Abcam, UK). Staining was performed using a DAB detection kit (Boster; Wuhan, China), with peroxidase activity and diaminobenzidine as a chromogen. Finally, the slides were sealed with a neutral balsam. Two independent researchers blindly examined the sections under an optical microscope (Leica Microsystems; Wetzlar, Germany) and assigned histopathological scores according to the McGuigan pathological standard [16]. Brown granules were considered immunohistochemically positive. For analysis, six fields at ×40 magnification were randomly selected, and the number of CD177^+^ neutrophils, stromal cells, and epithelial cells was counted, followed by the calculation of the average.

### Extraction of neutrophils from mouse bone marrow

Following euthanasia in mice, the skin was disinfected with 70% ethanol, and the femur and tibia were excised using scissors and forceps. The bones were placed in Dulbecco’s Modified Eagle Medium (DMEM, Thermo Fisher, USA) supplemented with 10% pre-cooled fetal bovine serum (FBS) and 1% double-antibody. The bone marrow cavity was flushed using a 10-mL syringe to collect the bone marrow cell suspension. The suspension was further filtered through a 100-µm cell strainer to remove impurities and debris. It was subsequently transferred to a 15-mL centrifuge tube and centrifuged at 427×*g* at 4 ^∘^C for 7 min, after which the supernatant was discarded. To lyse red blood cells, the cells were resuspended in 0.2% NaCl, followed by a rapid addition of a 1.6% NaCl solution for neutralization. The cells underwent another centrifugation at 427×*g* at 4 ^∘^C for 7 min and the supernatant was discarded. The cells were then resuspended in the complete medium and neutrophils were purified from the bone marrow cells using density gradient separation techniques.

### Co-culture of lung epithelial cells with neutrophils

We isolated neutrophils from the bone marrow of normal mice and subsequently stimulated them with LPS. The neutrophils were then treated with either IgG (Thermo Fisher, USA, 1 µg/mL), CD177 antibodies (Thermo Fisher, USA, 1 µg/mL), or left untreated. Simultaneously, a control group without LPS was established. After a 2-h treatment period, the neutrophils were co-cultured with LA-4 cells (Biotechnology, China) at a 1:1 ratio. The culture medium consisted of DMEM containing 10% FBS (HyClone, USA), penicillin (100 U/mL), and streptomycin (100 µg/mL), with the culture conditions set at 37 ^∘^C and 5% CO_2_. Four experimental groups were established: LPS alone, LPS+IgG, LPS+anti-CD177 monoclonal antibody, and blank control. After 24 h of culture, cells and supernatants were collected, and the levels of various inflammatory factors were detected using ELISA kits. Additionally, intracellular ROS and myeloperoxidase (MPO) levels were assessed using ROS (Beyotime, China) and MPO (Beijing Solarbio, China) kits.

### Determination of ROS and MPO levels

To assess changes in ROS and MPO levels, we employed assay kits from Beyotime (Beyotime, China) and Solarbio (Beijing Solarbio, China). Initially, we loaded the DCFH-DA probe (diluted 1:1000 in serum-free culture medium) into mouse lung tissue or neutrophils, followed by the measurement of lactate dehydrogenase absorbance at a wavelength of 460 nm using a microplate reader. For the MPO detection process, corresponding reagents were added to mouse lung tissue or neutrophils from the bone marrow after co-culture. Subsequently, ultrasonic disruption in an ice bath (200 W, 3 min) was performed, followed by centrifugation at 4 ^∘^C and 12,000 *g* for 10 min. The supernatant was collected for absorbance measurement at a wavelength of 460 nm using a microplate reader. The MPO activity was calculated using the following formula:


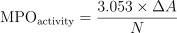
 or 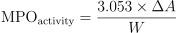, where *N* represents the cell number, *W* represents the sample weight, and ΔA represents the difference in absorption value measured by the experimental group minus the control group at 460 nm wavelength.

### Western blotting

The cell lysate was prepared by combining RIPA protein lysate (Servicebio, China) with PMSF (Servicebio, China) following ultrasound cracking of the homogenate. The protein concentration in the cell lysate was determined using a BCA protein concentration assay kit and calculating the protein loading for each well of the SDS-PAGE gel. Following electrophoresis, the protein was transferred to a PVDF membrane (Millipore, USA). The membrane was then blocked by incubating it with 10% defatted milk, followed by incubation with the corresponding primary antibody at 4 ^∘^C overnight. Subsequent steps included washing with TBST, incubation with the appropriate secondary antibody, and exposure to X-ray films after a final TBST wash. Antibodies that measure the expression of CD177 (Abcam, UK), inflammasome components [NLRP3 and Caspase-1 (both from Abcam, UK)], GAPDH (CST, USA), and peptidyl arginine deiminase (PAD4) (Thermo Fisher, USA) in neutrophils were employed in this experiment.

**Figure 1.**
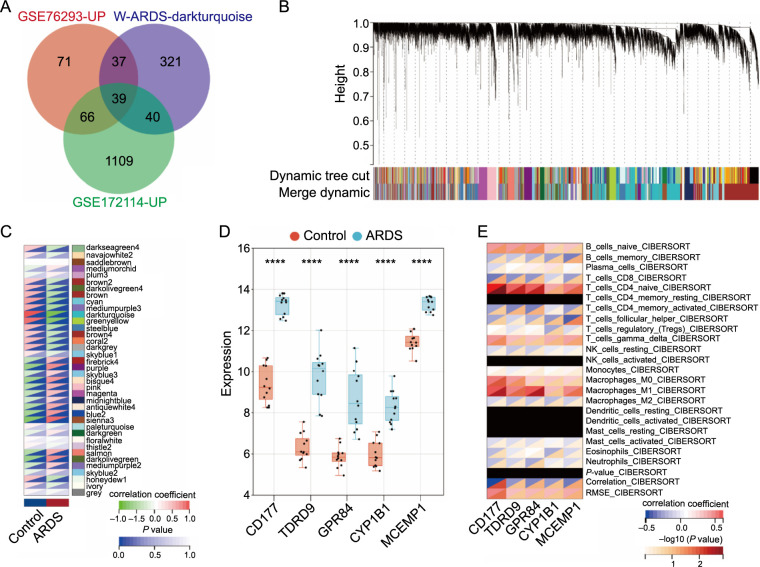
**Genes associated with ARDS.** (A) Venn analysis results; (B) WGCNA gene cluster map; (C) Heatmap of the module and phenotype correlation of WGCNA; (D) Box plots of five DEGs; (E) Matrix map of immune infiltration of the five genes based on the CIBERSORT method. ****P < 0.0001. ARDS: Acute respiratory distress syndrome; DEG: Differentially expressed gene.

### Ethical statement

This study was approved by the Laboratory Animal Management Committee of Jiangxi Province, China. All the experimental procedures involving animals were conducted in accordance with the Institutional Animal Care guidelines of Nanchang University, China, and approved by the Administrative Committee of Experimental Animals, Jiangxi Province, China (Approval No.: NCULAE-20221031161). This study also received approval from the Clinical Research Institutional Review Board of the Second Affiliated Hospital of Nanchang University and was conducted in accordance with the Declaration of Helsinki. Written informed consent was obtained from all participants before the study.

### Statistical analysis

We performed statistical analysis using GraphPad Prism 8.0 software (GraphPad, San Diego, CA, USA). We used *T*-test to analyze the mean difference between two groups of data and ANOVA to analyze the variance among three or more groups of data. For pairwise comparisons, we performed the least significant difference analysis as a post hoc test. Histogram data are presented as mean ± standard error (SEM). *P* < 0.05 indicates a statistically significant difference, denoted as *P < 0.05, **P < 0.01, ***P < 0.001, and ****P < 0.0001.

**Figure 2.**
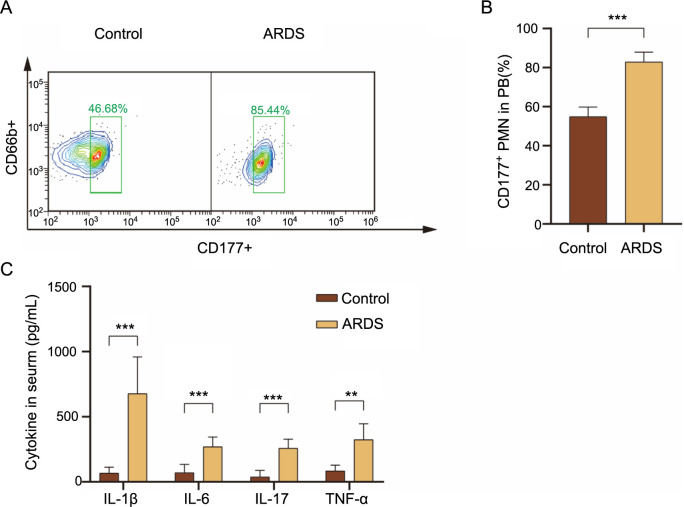
**CD177 expression in ARDS patients.** (A) Flow cytometry analysis illustrating the percentage of CD177+ neutrophils in peripheral blood; (B) Neutrophils were isolated from the peripheral blood of healthy volunteers (Control, *n* ═ 6) and ARDS patients (ARDS, *n* ═ 6). Flow cytometry was employed to detect CD66b+ neutrophils and CD177+ CD66b+ neutrophils; (C) The levels of common inflammatory factors in the peripheral blood of healthy controls and ARDS patients were measured using ELISA kits, following the manufacturer’s instructions. *P < 0.05, **P < 0.01, ***P < 0.001. ARDS: Acute respiratory distress syndrome; DEG: Differentially expressed gene; ELISA: Enzyme-linked immunosorbent assay.

## Results

### CD177 is a marker for the development of ARDS/ALI

**Figure S1.**
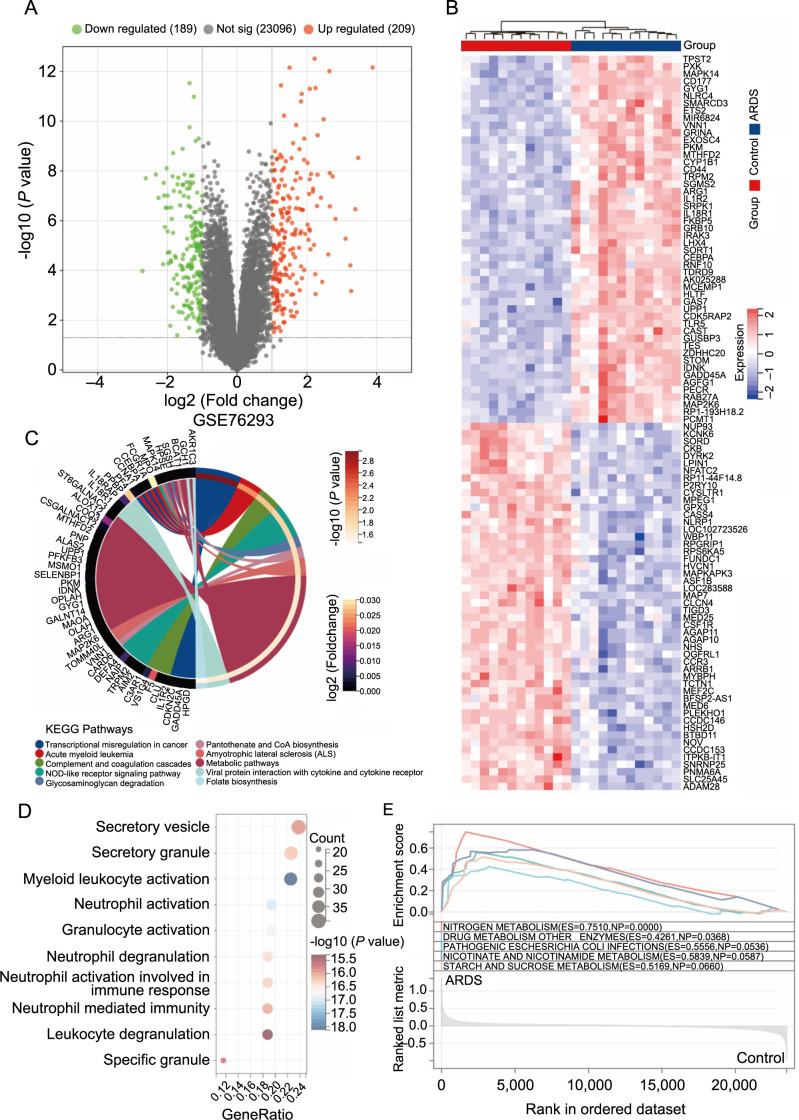
**Bioinformatical analysis of ARDS occurrence.** (A) Volcano map of differentially expressed genes in GSE76293; (B) Thermal clustering map of limma; (C and D) Significantly differentially expressed genes enriched in GO and KEGG pathways; (E) Gene set enrichment analysis showed that ARDS markers were enriched in inflammatory signaling pathways, apoptosis pathways, and immune-related processes. ARDS: Acute respiratory distress syndrome; GO: Gene ontology; KEGG: Kyoto Encyclopedia of Genes and Genomes.

ARDS is a life-threatening respiratory condition marked by bilateral pulmonary consolidation and severe hypoxemia caused by non-cardiogenic pulmonary edema. This condition leads to respiratory failure and high mortality rates [17]. In our endeavor to swiftly pinpoint genes intimately linked to the onset and progression of ARDS, we methodically scrutinized genes associated with ARDS in two transcriptome datasets (GSE76293 and GSE172114) from the GEO public database. Employing gene differential and WGCNA analysis, we identified 39 DEGs significantly associated with ARDS (Figure 1A–1C). These genes are primarily involved in key biological processes, such as immune regulation, cell apoptosis, and the inflammatory response. Further functional enrichment analysis underscored their roles in inflammatory signaling pathways, cell apoptosis pathways, and immune-related processes (Figure S1). Among the 39 DEGs, we focused on the five most variably expressed genes: *CD177*, *TDRD9*, *GPR84*, *CYP1B1*, and *MCEMP1*. To delve deeper into the functions and interrelationships of these five genes, we analyzed immune infiltration and gene expression diagrams (Figure 1C and 1D). The results revealed that the *CD177* gene significantly influences ARDS development, suggesting its crucial role as a pathogenic factor in ALI.

### The rate of CD177^+^ neutrophils was significantly upregulated in the peripheral blood of ALI patients

CD177, a specific protein located on the membrane of neutrophils, is anchored to the cell membrane through glycolipid-phosphatidylinositol and significantly influences neutrophil activation and migration [18]. Notably, CD177 overexpression has been observed in the tissues of patients with inflammatory bowel disease [11] and colorectal cancer [19]. In our study, we identified CD177 as a gene associated with ARDS or ALI using bioinformatics methods. To explore CD177’s role in ARDS, we collected peripheral blood samples from ARDS patients and healthy volunteers. We then analyzed the proportion of CD177+ cells among CD66b+ neutrophils using flow cytometry. Our results revealed a significant increase in the proportion of CD177+ neutrophils in the peripheral blood of ARDS patients compared to healthy volunteers (Figure 2A and 2B). Furthermore, we assessed the levels of common inflammatory factors in patient peripheral blood using ELISA kits (Figure 2C). This analysis showed a marked increase in inflammatory factor levels in ARDS patients. These findings suggest that CD177 plays a crucial role in the onset and progression of ARDS.

**Figure 3.**
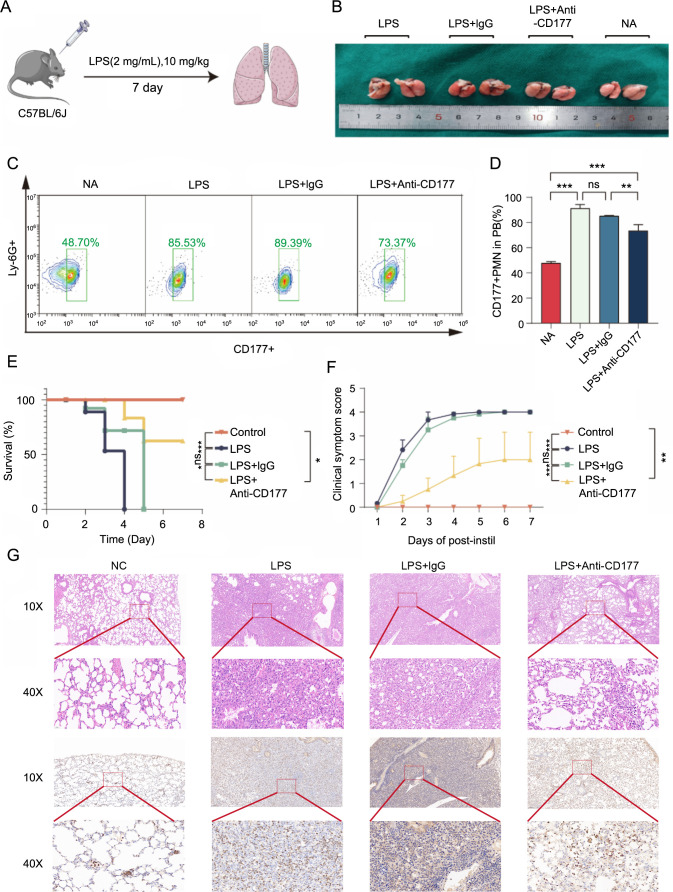
**CD177 inhibition can attenuate the acute inflammatory response of LPS-induced ALI**. We established a mouse model of lung injury with LPS (A) and treated it with anti-CD177 antibody (0.1 ng) by intraperitoneal injection, with IgG antibody as a control (0.1 ng). After 48 h, we observed a significant reduction in pulmonary inflammation (B), along with a lower percentage of CD177+ neutrophils in peripheral blood (C and D), improved survival rate (E), and clinical symptom score (F) in mice. Lung tissue sections also showed the difference between the LPS and anti-CD177 antibody treatment groups (G). *P < 0.05, **P < 0.01, ***P < 0.001. LPS: Lipopolysaccharide; ALI: Acute lung injury.

**Figure 4.**
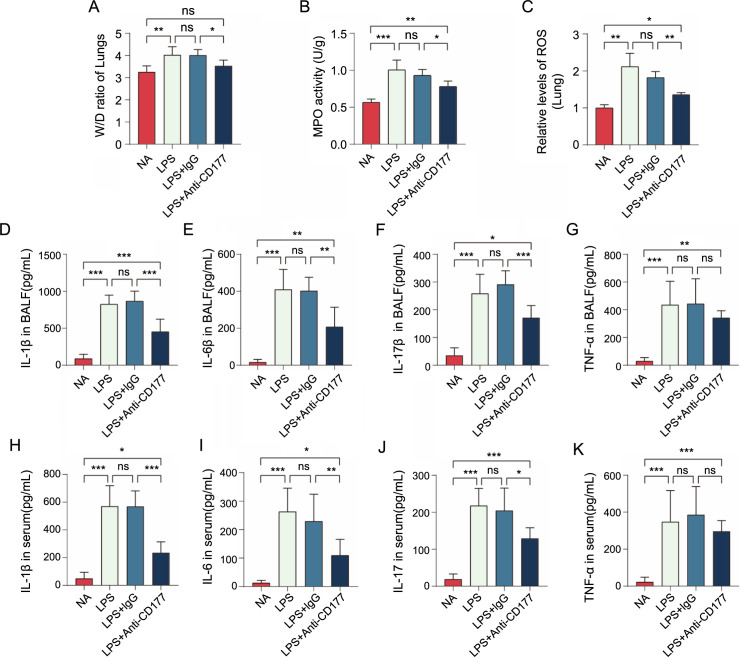
**CD177 plays an important mediating role in LPS-induced ALI.** (A) Blocking the CD177 antigen on the neutrophil surface reduces pulmonary edema; (B) diminishes alveolar neutrophil infiltration, lung tissue MPO levels; (C) and ROS levels; (D–G) After 48 h of establishing the mouse ALI model, levels of common inflammatory factors in the BALF and (H–K) PB were measured using ELISA kits, according to the manufacturer’s instructions. *P < 0.05, **P < 0.01, ***P < 0.001. LPS: Lipopolysaccharide; ALI: Acute lung injury; BALF: Bronchoalveolar lavage fluid; ELISA: Enzyme-linked immunosorbent assay.

### Modulation of CD177 alleviates LPS-induced ALI in C57BL/6J mice

To further elucidate the relationship between CD177 expression and ALI, C57BL/6J strain mice were employed as experimental models. An ALI model was created through intratracheal injection of LPS at a concentration of 10 mg/kg, while PBS was used as a control. Due to the unavailability of commercially available CD177 agonists and inhibitors, a CD177 monoclonal antibody and an IgG antibody were utilized to modulate CD177 function and assess its impact on ARDS/ALI in mice. LPS, known for inducing ALI, leads to severe respiratory distress, rapid onset, and high mortality in mice, often resulting in death within 2–4 days following LPS administration. Surprisingly, when CD177 was targeted using monoclonal antibodies, we observed reduced pulmonary edema and a decrease in the proportion of CD177-positive cells in mice (Figure 3A–3D). Further detailed investigation showed significant improvements in clinical manifestations and lung tissue damage in the mice, along with a noticeable increase in survival rates (Figure 3E and 3F). Histological alterations in the ALI model were examined using hematoxylin and eosin staining. Compared to the LPS control group, treatment with the anti-CD177 antibody significantly reduced alveolar interstitial edema and the infiltration of inflammatory cells (Figure 3G). The anti-CD177 antibody exhibited a substantial improvement in conditions like pulmonary edema, thickening of the alveolar wall, and neutrophil infiltration in mice.

**Figure 5.**
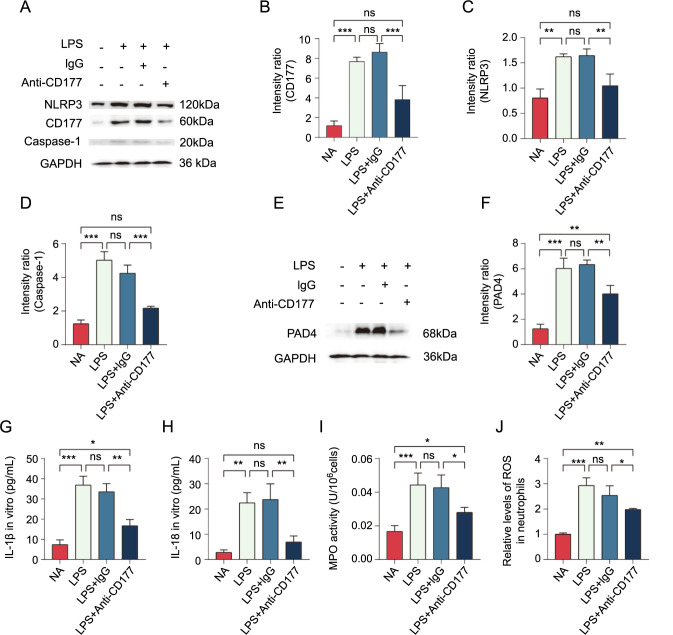
**Impact of CD177 on the formation and release of the inflammasomes in neutrophils.** (A–D) We measured the levels of inflammasome-related proteins NLRP3 and caspase-1; (E and F) Based on the WB method, we detected the PAD4 expression levels, a marker of NETs; (G and H) We used ELISA kits to measure the concentrations of inflammatory factors in the supernatants; (I and J) We used ROS and MPO kits to measure the relative levels of MPO and ROS in the cells. *P < 0.05, **P < 0.01, ***P < 0.001. NET: Neutrophil extracellular trap; ELISA: Enzyme-linked immunosorbent assay; ROS: Reactive oxygen species; MPO: Myeloperoxidase.

### In the ALI model, CD177 antibody blocked the activation of neutrophils

Our research findings underscore the pivotal role of CD177 in the pathogenesis of ALI. We calculated the wet/dry ratio and measured the levels of ROS and MPO in the lung tissues of different groups. Inhibiting CD177 function on neutrophils effectively reduced the wet/dry ratio, as well as MPO and ROS levels in lung tissue induced by LPS (Figure 4A–4C). Furthermore, we evaluated the levels of several inflammatory cytokines in peripheral blood and BALF and, surprisingly, discovered a statistically significant synchronous decrease in the levels of IL-6, IL-1β, IL-17, and TNF-α in both peripheral blood and BALF (Figure 4D–4K). These findings indicate that CD177 affects the severity of lung injury in ARDS by influencing neutrophil activation and the release of inflammatory cytokines. Therefore, treatment targeting CD177 may inhibit excessive neutrophil activation, thus mitigating inflammatory injury.

### CD177 mediates the formation of the NLRP3 inflammasomes

**Figure S2.**
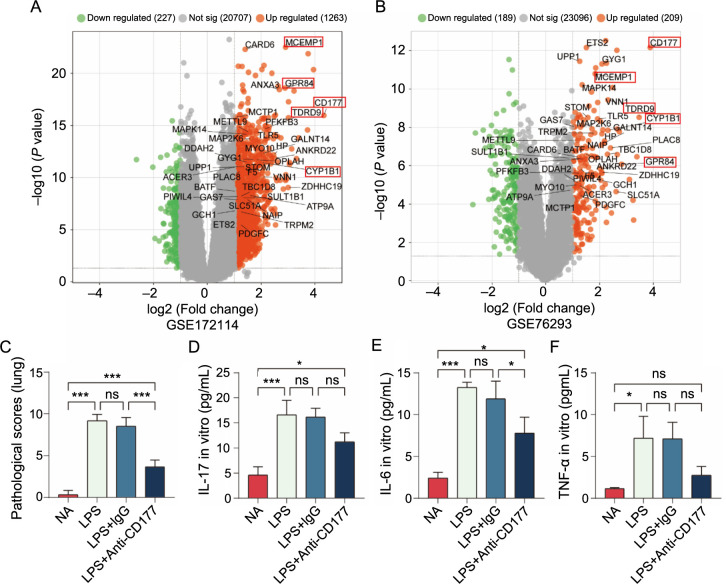
(A and B) Volcano map of GSE172114 and GSE76293; (C) Pathological score of lung tissue in LPS-induced ALI model mice; (D–F) IL-17, IL-6, and TNF-α levels measured after co-culture of neutrophils and lung epithelial cells. *P < 0.05, **P < 0.01, ***P < 0.001. LPS: Lipopolysaccharide; ALI: Acute lung injury.

To further explore the molecular mechanism of CD177-induced lung injury in the ALI model, we developed a co-culture system of pulmonary epithelial cells and neutrophils to mimic pulmonary inflammation. Neutrophils, extracted from the bone marrow of normal mice, were co-cultured with pulmonary epithelial cells at a 1:1 ratio. We then treated four groups of cells with LPS, LPS+IgG, LPS+anti-CD177, and PBS, incubating them at 37 ^∘^C. By employing the ELISA method, we measured the inflammatory cytokine levels in the culture medium. We found that anti-CD177 effectively reduced neutrophil activation and inhibited the secretion of inflammatory cytokines. Notably, the most significant reductions were observed in IL-1β and IL-18 levels (Figure 5G and 5H), while IL-6, IL-17, and TNF-α levels also exhibited varying degrees of decrease (Figure S2). According to the existing literature, IL-1β secretion levels can reflect the activation degree of the NLRP3 inflammasome [20, 21]. We analyzed the expression levels of NLRP3 and caspase-1 in the four cell groups using Western blotting. The findings showed that the LPS and LPS+IgG groups exhibited significantly elevated expressions of NLRP3 and caspase-1, while anti-CD177 effectively reduced their expression (Figure 5A–5D). In addition, anti-CD177 markedly lowered the expression of PAD4 (Figure 5E and 5F), a marker of NETs, as well as the relative concentrations of MPO (Figure 5I) and ROS (Figure 5J) in the cells, aligning with the results from the mouse model. These findings suggest that LPS can activate the NLRP3 inflammasome through the CD177-mediated signaling pathway, thereby exacerbating pulmonary inflammation injury.

## Discussion

ALI and ARDS represent a group of pulmonary disorders clinically marked by acute hypoxemic respiratory failure, commonly encountered in intensive care units. Their onset stems from damage to the alveolar-capillary membrane, resulting in fluid leakage into the alveoli and reduced lung compliance. Patients typically present with symptoms like coughing, difficulty breathing, and cyanosis, often requiring mechanical ventilation support [10, 22]. The diagnostic criteria for ALI and ARDS are based on the PaO_2_/FiO_2_ ratio, chest imaging, and the cause of pulmonary edema [14]. However, there is currently no specific medication targeting the excessive inflammatory response in ARDS. The prognosis for ALI and ARDS varies depending on the severity and associated complications, with a mortality rate of approximately 30%–40%. Survivors of these conditions may also suffer from chronic lung function impairment [23].

Neutrophils are crucial as a key pathological factor in ARDS/ALI, accumulating in substantial numbers around pulmonary vessels and bronchi, and actively engaging in the inflammatory response [24, 25]. Upon exposure to exogenous or endogenous stimuli, neutrophils generate a large amount of ROS, NETs, and calprotectin, and other effector molecules within the pulmonary interstitium and alveoli. This production is catalyzed by NADPH oxidase following phagocytosis or interaction with pathogens. Concurrently, they persist in activating and recruiting additional neutrophils [26, 27]. These molecules serve not only to combat pathogens but also to amplify the inflammatory response, increase vascular permeability, induce fluid and protein exudation, foster pulmonary edema, and cause lung tissue damage. Therefore, therapeutic strategies targeting the excessive activation of neutrophils and the formation of NETs offer the potential for alleviating severe symptoms and improving the prognosis of ARDS [28, 29].

Our research identifies CD177 as a molecular marker linked to the severity of ALI/ARDS. Utilizing bioinformatics and flow cytometry, we found a notably higher proportion of CD177+ neutrophils in ARDS patients compared to healthy controls. CD177, expressed exclusively in a subgroup of neutrophils, accounts for approximately 45%–65% of circulating cells [18, 30]. Recent studies have confirmed that the CD177+ neutrophil subgroup in inflammatory bowel disease can release substantial amounts of ROS and NETs, establishing a strong antibacterial barrier [11]. ROS and NETs, capable of neutralizing or immobilizing pathogens, prevent their spread and enhance neutrophils’ phagocytic function. Their production is dependent on the activation of NADPH oxidase, alongside the release of elastase and MPO from neutrophil granules. NETs primarily comprise a network structure containing DNA, histones, granular proteins, and enzymes, such as CitH3, MPO, and MMP-9 [31, 32]. Our findings suggest that in the ALI model, the CD177 antibody can reduce the levels of inflammatory factors, MPO, and ROS in mouse peripheral blood and BALF, thereby alleviating the inflammatory damage associated with ALI. This indicates that anti-CD177 could effectively reduce neutrophil activation. Moreover, Zhang et al. [12] investigated the protective effect of exogenous CD177 protein in acute pancreatitis mice and reported a significant reduction in pathological damage to the pancreas and lungs, improving survival rates. Our study mirrors these findings, suggesting that exogenous CD177 protein could be a potential therapeutic agent for ARDS, similar to CD177 antibodies. Its mechanism may involve CD177’s regulation of neutrophil activation and NET formation, although the specific signaling pathway involved warrants further investigation.

**Figure 6.**
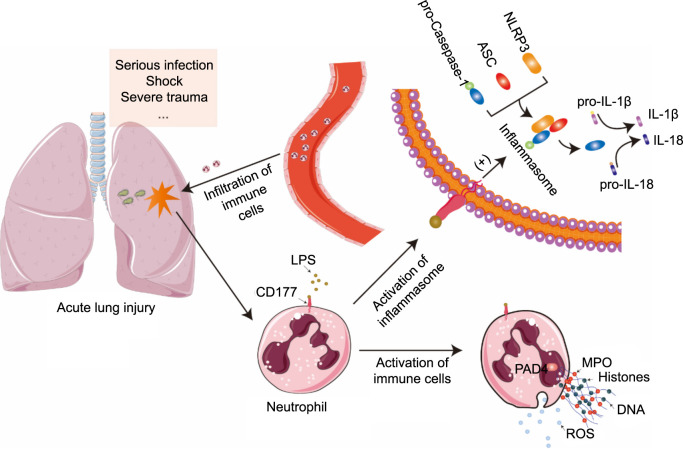
**Mechanistic diagram of CD177 in neutrophils.** ROS: Reactive oxygen species; MPO: Myeloperoxidase; LPS: Lipopolysaccharide; DNA: Deoxyribonucleic acid.

It is noteworthy that intervention in the ALI model with anti-CD177 led to the most significant reduction in IL-1β levels in peripheral blood and BALF compared to other inflammatory cell factor levels. This indicates that IL-1β may act as a specific effector molecule in the CD177 signaling pathway and serves as an effector molecule of the NLRP3 inflammasome [33, 34]. This finding suggests that CD177 is an activation pathway for the NLRP3 inflammasome (Figure 6). The inflammasome is a multi-protein complex primarily composed of NLRP3, caspase-1, and ASC protein [35]. When cells are challenged by viruses or bacteria, NLRP3 recruits the ASC protein, activates caspase-1, and induces the maturation and release of inflammatory factors, including IL-1β [20, 36]. Based on the results from the mouse lung injury model, we hypothesize that CD177 positively influences the activation of NLRP3 inflammasomes, leading to increased IL-1β release and intensifying the severity of lung injury. To corroborate this hypothesis, we further examined the effects of IgG antibodies and CD177 antibodies on the co-culture system of lung epithelial cells and neutrophils, particularly focusing on changes in inflammasome-related proteins such as NLRP3, ASC, and caspase-1. Our findings demonstrate that CD177 antibodies significantly reduce the expression levels of NLRP3 and caspase-1 in neutrophils cultured in vitro. These results suggest that CD177 facilitates the activation of NLRP3 inflammasomes.

There are three limitations to this study. First, this study only preliminarily explored the correlation between CD177 and the NLRP3 inflammasome but did not unveil its specific mechanism of action. To further validate the hypotheses and findings of this study, more accurate verification of the correlation between CD177 and ARDS is needed, such as using CD177^−/−^ mice. Second, to further validate the targeted therapeutic role of CD177, more animal experiments and clinical trials are needed to enhance the safety and effectiveness of its treatment. Finally, the mouse ALI model induced by LPS is a widely used experimental method, but it cannot fully capture all the characteristics of human ARDS. Human ARDS has multiple causes, and for our experiment, we selected patients with intra- and extrapulmonary infections as an inclusion criterion to approximate the credibility of the model. Therefore, we hope that future research can utilize animal models that are more representative of human ARDS.

## Conclusion

In conclusion, our research highlights the critical role of CD177 in neutrophil activation, the release of NETs, and the regulation of the NLRP3 inflammasomes. CD177 exhibits dual effects: it displays anti-inflammatory protective qualities as well as pro-inflammatory damaging properties, which may contribute to an imbalance in immune homeostasis. As an indicator of neutrophil activity and the level of inflammatory response, CD177 is not only involved in the pathogenesis of ARDS/ALI but also emerges as a promising therapeutic target. Modulating its expression or function could offer significant potential in enhancing the prognosis of patients with ARDS.

## Supplemental data

**Figure S3.**
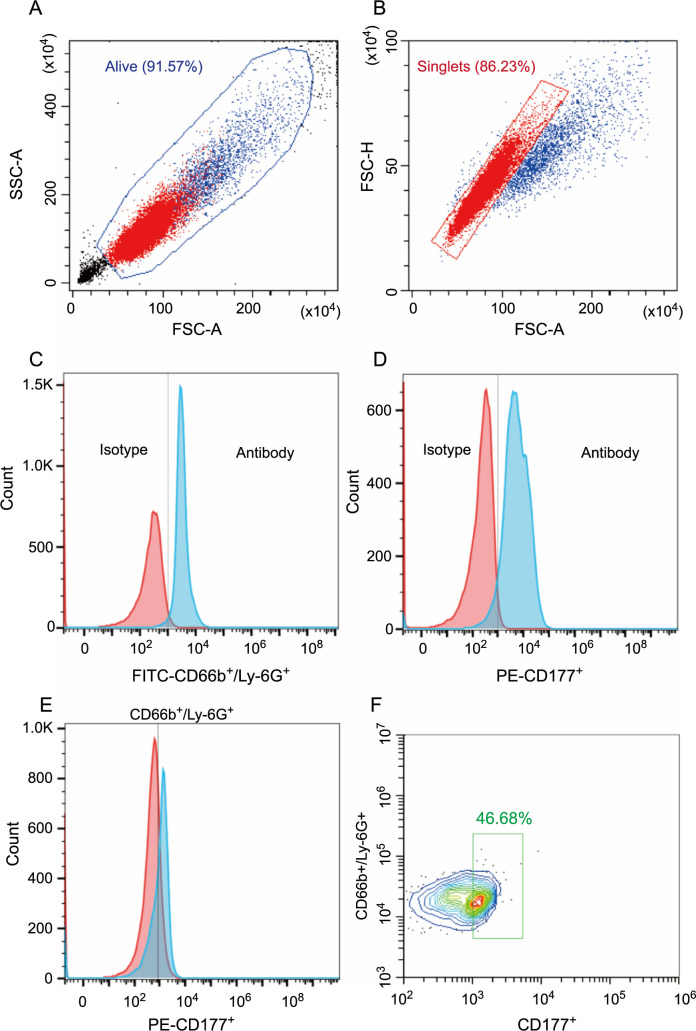
**Flow cytometry gating strategy.** (A) We delineated live cells, excluded dead cells and cell debris; (B) and then removed the adhesions; (C) The fluorescence thresholds of FITC and (D) PE were set using Isotype Control antibodies IgG; (E) After FITC-CD66b^+^/Ly-6G^+^ neutrophils were isolated by flow sorting, PE-CD177^+^ fluorescent antibody was used to (F) further detect the CD177^+^ neutrophils ratio.

## Data Availability

The data that supports the findings of this study is available upon a reasonable request from the corresponding and first authors.
